# Editorial Comment: The Impact of Ureteral Access Sheath Use on the Development of Abnormal Postoperative Upper Tract Imaging after Ureteroscopy

**DOI:** 10.1590/S1677-5538.IBJU.2021.02.06

**Published:** 2021-02-03

**Authors:** Alexandre Danilovic

**Affiliations:** 1 USP Faculdade de Medicina Hospital das Clínicas São Paulo SP Brasil Serviço de Urologia, Hospital das Clínicas da Faculdade de Medicina da USP - HCFMUSP, São Paulo, SP, Brasil

John L Cooper ^1^, Nathaly François ^1^, Michael W Sourial ^1^, Hiroko Miyagi ^2^, Justin R Rose ^1^, John Shields ^3^, et al.

J Urol. 2020 Nov;204(5):976-981.

## COMMENT

The ureteral access sheath (UAS) is a commonly used disposable device in flexible ureteroscopy (fURS) (1). Use of UAS in fURS is reported to improve visibility, reduce intrapelvic pressure and increase efficiency of the procedure. On the other hand, the use of this device may cause ureteral injury during its placement or while it is already placed due to ureteral ischemia (2, 3).

This retrospective study investigated the association of UAS use during 1332 ureteroscopic procedures with abnormal post-URS imaging. UAS (12/14F in 95.7%) was used in 78% of the cases. Upper tract imaging with KUB, ultrasound or CT scan was obtained at eight-week follow-up visit after URS. No significant association was seem between use of UAS and abnormal post-URS imaging. Incidence of hydronephrosis was 12% and the ureteral stricture rate was only 0.66%.

Besides the main finding of no significant association between the use of UAS and development of abnormal post-URS imaging, this study brings light on the value of routine follow-up imaging following URS. There is no specific recommendation coming from guidelines about modality or timing of imaging after fURS. Follow-up imaging after ureteroscopy is essential due to irreversible renal function impairment caused by silent ureteral stones and to check for complications (4, 5). Non-contrast CT scan is the gold standard imaging after fURS for both stone free rate and complications but ultrasound is very efficient for looking for complications and residual stone fragments > 2 mm (6).
